# 磁性一步式净化-高效液相色谱-三重四极杆/复合线性离子阱质谱测定鲤鱼中54种兽药残留

**DOI:** 10.3724/SP.J.1123.2024.12010

**Published:** 2025-11-08

**Authors:** Yupeng WEN, Wendi HUO, Chaoying ZHANG, Huan LIU, Yingchun MU, Lidong WU, Jincheng LI

**Affiliations:** 1.上海海洋大学水产与生命学院，上海 201306; 1. College of Fisheries and Life Science，Shanghai Ocean University，Shanghai 201306，China; 2.中国水产科学研究院，农业农村部水产品质量安全控制 重点实验室，北京 100141; 2. Key Laboratory of Control of Quality and Safety for Aquatic Products，Ministry of Agriculture and Rural Affairs，Chinese Academy of Fishery Sciences，Beijing 100141，China; 3.北京石油化工学院新材料与化工学院，北京 102617; 3. School of New Materials and Chemical Engineering，Beijing Institute of Petrochemical Technology，Beijing 102617，China

**Keywords:** 兽药, 磁性一步式净化, 高效液相色谱-三重四极杆/复合线性离子阱质谱, 鲤鱼, veterinary drugs, magnetic one-step purification, high performance liquid chromatography-triple quadrupole/composite linear ion trap mass spectrometry （HPLC-QTRAP-MS/MS）, crap

## Abstract

水产食品中含有脂肪、蛋白质等干扰物质，这些干扰物质不仅会影响目标化合物分析检测的结果，大大降低检测效率，也会对检测仪器造成污染。干扰物质的有效去除是食品质量检测领域的关键环节之一。为了降低水产品中干扰物质的影响，本研究采用磁性一步式净化，结合高效液相色谱-三重四极杆/复合线性离子阱质谱（HPLC-QTRAP-MS/MS）建立了鲤鱼中54种兽药残留的检测方法。对样品前处理过程进行优化，添加一定量的四氧化三铁颗粒使得净化材料获得磁性，以金属有机骨架材料（ZIF-67）作为去除提取液中的脂肪酸和有机酸等干扰物质的净化剂，以十八烷基键合硅胶净化材料（C_18_）作为去除非极性杂质的辅助净化剂材料，采用磁性一步式净化进行净化处理；借助超高效液相色谱实现快速分离，三重四极杆质谱仪进行测定，基质匹配外标法定量分析，实现快速、准确测定鲤鱼中54种兽药残留量。54种兽药在0.5~50.0 µg/L范围内线性关系良好，相关系数（*r* ^2^）均在0.99以上，其检出限和定量限分别为0.5~1.0 µg/kg和1.0~2.0 µg/kg；在2.0、5.0、20.0 µg/kg 3个水平下，样品加标回收率为81.34%~109.85%，精密度的考察结果显示日内及日间相对标准偏差均小于10%。该方法可作为鲤鱼中兽药监测方法，其符合经济、简便、高效的要求，且灵敏度高，重复性好。

## Abstract (en)

Aquatic food products contain interfering substances， such as fats and proteins， which affect the analysis and detection of target compounds by significantly reducing detection efficiency and contaminating detection instruments. Effectively removing interfering substances is a key step in the field of food quality testing. To reduce the influence of interfering substances in aquatic products， this study established a detection method for 54 veterinary drug residues in carp by using magnetic one-step purification combined with high performance liquid chromatography-triple quadrupole/composite linear ion trap mass spectrometry （HPLC-QTRAP-MS/MS）. The purification process uses solvothermally synthesized spherical magnetite as the magnetic material. This material is strongly magnetic and enables impurities to be efficiently separated and collected. Additionally， a metal-organic framework material with an amino-rich surface （ZIF-67） was self-assembled to serve as the purifying agent. ZIF-67 is particularly effective at removing polar impurities during the magnetic one-step purification process. Scanning electron microscopy confirmed the successful synthesis of both materials， ensuring their structural integrity and suitability for the intended application.Acetonitrile was chosen as the extraction solvent owing to its excellent solubilizing properties. Furthermore， formic acid was added to enhance the extraction efficiencies of acidic veterinary drugs. Anhydrous sodium sulfate was used to remove water from the matrix， thereby further improving the purity of the extract. The extract underwent magnetic one-step purification following centrifugation， which effectively removed interfering substances while preserving the target compounds. Chromatographic separation was achieved using the C_18 _RRHD column （15 cm×4.6 mm， 2.7 µm）， with 5 mmol/L ammonium acetate solution （containing 0.2% formic acid） and 0.2% formic acid in methanol used as mobile phases. HPLC-QTRAP-MS/MS was then used to separate and determine the analytes， with data collected in multiple reaction monitoring （MRM） mode. The 54 veterinary drugs were quantitatively analyzed using matrix-matched standard curves， thereby ensuring accurate quantification even for complex matrices. Various parameters that affect the purification fillers were investigated to optimize the purification process. Octadecyl-bonded silica gel （C_18_） was used as an auxiliary purification material as it is known to effectively remove non-polar impurities. Combining 60 mg of ZIF-67 with 50 mg of C_18_ yielded the best purification results. All 54 compounds exhibited weak matrix effects after pretreatment and purification， with a value of only 18.11% recorded for the compound that exhibited the most significant matrix effect. The 54 veterinary drugs exhibited excellent linear relationships between response and concentration in the 0.5–50.0 µg/L range， with all coefficients of determination （*r*²） above 0.99. Detection and quantification limits of 0.5–1.0 and 1.0–2.0 µg/kg were recorded， respectively. The performance of the method was evaluated by spiking blank carp matrix samples at three concentrations （2.0， 5.0， and 20.0 µg/kg）. The recovery rates of the spiked samples ranged between 81.34% and 109.85%， and intra-day and inter-day relative standard deviations were less than 10%. The rapidity and accuracy of the developed method were assessed through comparisons with existing veterinary-drug detection methods， which revealed that the developed method is more sensitive and repeatable. Furthermore， the feasibility of the established detection method was verified by testing commercial aquatic products. No target veterinary drugs were detected in actual samples， which highlights the reliability of the developed method. In conclusion， the method developed in this study provides an effective means of monitoring veterinary drugs in carp. It meets the requirements of economy， simplicity， and efficiency while exhibiting high sensitivity and reproducibility. The developed approach represents a significant advancement in the field of aquatic food safety testing and is expected to ensure more accurate and reliable detection of veterinary drug residues.

随着我国经济的快速发展，居民在饮食健康方面的关注度也在不断提升。水产品因其营养丰富、低脂肪和高蛋白的特点，受到消费者的青睐。但是，个别养殖商家对合理用药缺乏足够的认识，其在防治动物病害的过程中，会有误用兽药或过量使用的操作，导致水产食品中兽药残留超标。这不仅直接影响水产动物的产品质量以及渔业的健康发展，还能通过食物链的传递作用间接影响人民群众的健康。我国《食品安全国家标准 食品中兽药最大残留限量》（GB 31650-2019）、《食品安全国家标准 食品中41种兽药最大残留限量》（GB 31650.1-2022）和中华人民共和国农业农村部公告第250号等标准和规范中对类固醇激素类、硝基咪唑类等药物的限量也进行了严格规定^［1，2］^。因此，为了促进水产行业的高质量发展，开展水产品中兽药残留检测方法研究已经成为行业热点。

目前，水产品中兽药残留的检测方法主要有高效液相色谱法^［3］^、高效液相色谱-质谱法^［4］^、气相色谱法^［5］^等。其中，高效液相色谱-质谱法凭借其灵敏度高、定性和定量准确的优点，成为目前检测水产品中兽药残留的主流方法。

研究人员虽然一直致力于发展水产品中兽药残留的检测方法，但由于水产品中含有较高含量的脂肪和蛋白质等干扰物质，因此仍需要采用复杂的样品前处理方法，如固相萃取^［6］^、QuEChERS^［7］^和磁性一步式净化方法^［8］^等，进行提取液中目标化合物的净化、富集。磁性一步式净化方法凭借其简单、快速的优点，引起了关注。

磁性一步式净化是在传统QuEChERS萃取和分散固相萃取除杂净化的基础上发展而来的一种新型高效样品前处理技术^［9］^。传统的QuEChERS萃取和分散固相萃取除杂净化均需采用高速离心以实现提取液与样品基质或净化填料的分离，无法用于大量样品的快速前处理。崔勇等^［10］^发现在分散固相萃取除杂净化的过程中，将磁性功能材料作为净化吸附剂，可以快速完成净化液和样品基质的快速分离，建立了磁性一步式净化-超高效液相色谱-质谱测定*α*-鹅膏毒肽的方法。刘真真等^［11］^利用磁性纳米材料Fe_3_O_4_-PSA磁分离和净化的优势与超高效液相色谱-三重四极杆质谱（UPLC-MS/MS）定量分析的优势，建立了同时对猕猴桃中52种农药及相关农药代谢物进行快速分析的方法。除了磁性纳米材料，刘小琦等^［12］^在磁性一步式净化中，将磁性纳米颗粒和PSA作为混合净化吸附剂以增强净化效果，建立了鱼中13种全氟及多氟烷基化合物高效液相色谱-质谱分析方法，加标回收率为78.1%～118%。磁性材料是近年国内外研究的热点，其具有超顺磁性、易功能化修饰、易分离目标分析物等优势^［13，14］^。在含有目标分析物的提取液中，将磁性净化材料直接添加到其中，分散的磁性净化材料会将提取液中的杂质吸附到表面；在外部磁力作用下，杂质可快速随材料聚集，从而实现高效的固液分离。这一过程省略了耗时的过滤或离心操作，显著提升了分离效率。在磁性材料的作用下，不仅净化效率得到提高，而且可以实现短时内处理高通量样品的目的。本研究通过自组装合成了表面富含氨基的金属有机骨架材料（ZIF-67）^［15］^，并选择其作为磁性一步式净化过程中极性杂质的净化剂。ZIF-67由过渡金属和有机咪唑脂反应形成，其表面含有氨基，可通过离子交换作用去除提取液中的脂肪酸和有机酸等干扰物质^［16］^。为了进一步增强净化效果，在磁性四氧化三铁、ZIF-67的基础上，以十八烷基键合硅胶净化材料（C_18_）作为去除非极性杂质的辅助净化剂，从而获得效果优良的磁性一步式净化材料。基于此，本研究采用高效液相色谱-三重四极杆/复合线性离子阱质谱（HPLC-QTRAP-MS/MS），以水产品中54种兽药残留为目标，以鲤鱼为研究对象，通过结合ZIF-67的磁性一步式净化方法，建立了鲤鱼中54种兽药的检测方法，为鲤鱼等鱼类产品中兽药残留的快速检测提供了技术支撑，也为其他种类水产品的兽药残留检测方法的开发提供了理论依据。

## 1 实验部分

### 1.1 仪器、试剂与材料

QTRAP^® ^5500 高效液相色谱-三重四极杆/复合线性离子阱质谱仪（美国SCIEX公司）；JC-FA3204电子天平（青岛精诚仪器仪表有限公司）；45 Position N-EVAP氮吹仪（美国Organomation Aossociates公司）；0.22 μm Nylon针式过滤器（温州迈凯科技有限公司）；L18-P631多功能粉碎机（浙江红素实业有限公司）；JL-PO2500S多管振荡混匀仪（上海靳澜仪器制造有限公司）；50 mL水热合成高压反应釜（上海凌科实业发展有限公司）。

乙腈（HPLC级）和甲醇（HPLC级）均购自美国Baker公司；甲酸（分析纯）购自北京化工厂；乙酸铵（色谱级，99.0%）、无水硫酸钠（分析纯，99.00%）、乙二醇（分析纯，98%）、FeCl_3_·6H_2_O（分析纯，99%）和Co（NO_3_）_2_·6H_2_O（分析纯，99%）均购自上海阿拉丁生物科技股份有限公司；2-甲基咪唑（分析纯，99%）购自上海麦克林生化科技股份有限公司；C_18_（40~60 μm）购自上海安谱实验科技股份有限公司；蒸馏水（杭州娃哈哈集团有限公司）；54种兽药混合标准溶液（100 mg/L）购自天津阿尔塔科技有限公司。鲤鱼样品购自北京市本地市场。

### 1.2 标准溶液的配制

溶剂混合标准工作溶液：以甲醇为溶剂，将54种目标化合物的标准储备溶液（100 mg/L）进行逐级稀释，得到质量浓度为1 mg/L的目标化合物混合标准工作液，配好后于-20 ℃避光保存。

基质混合标准溶液：将带皮肌肉经过前处理操作，得到空白基质提取液，对上述混合标准工作液进行稀释，得到质量浓度为0.5~50 μg/L的系列基质匹配混合标准溶液。

### 1.3 磁性颗粒的制备

以溶剂热法在高温髙压条件下合成了球形四氧化三铁，具体步骤：以FeCl_3_·6H_2_O（3.06 g）、无水乙酸钠（3.60 g）和乙二醇（80 mL）为原料，将混合液进行30 min的磁力搅拌后，转移至50 mL高压反应釜内。然后，将最大承压能力为2 MPa的反应釜放置于烘箱中，烘箱内部的温度控制在200 ℃。在此条件下，进行12 h的反应。

### 1.4 ZIF-67的制备

在100 mL蒸馏水中，倒入4 g Co（NO_3_）_2_·6H_2_O，搅拌溶液使其溶解混匀；在质量浓度为15 g/L的400 mL 2-甲基咪唑溶液中，将硝酸钴溶液缓缓倒入。然后将混合物继续进行12 min的搅拌；60 ℃烘箱中放置20 h后，生成紫色晶体。

### 1.5 样品制备方法

#### 1.5.1 提取

取冷冻保存的鲤鱼样品解冻至室温，称取（2.00±0.01） g样品于15 mL离心管中。然后，加入10.0 mL 1%甲酸乙腈，在2 500 r/min的条件下涡旋振荡10 min；称取3 g无水硫酸钠加入离心管，再次涡旋振荡10 min；在8 000 r/min的转速条件下离心5 min；然后取上层清液至15 mL离心管中，待净化。

#### 1.5.2 净化

移取2 mL上清液于5 mL离心管中（含有200 mg磁性颗粒、100 mg无水硫酸钠、60 mg ZIF-67和50 mg C_18_）；涡旋振荡1 min，磁分离；取上清液，氮吹浓缩后，复溶；过0.22 μm滤膜，待上机检测。

### 1.6 分析条件

#### 1.6.1 色谱条件

色谱柱：C_18 _RRHD色谱柱（15 cm×4.6 mm，2.7 µm）；柱温箱柱温为45 ℃；流速为0.40 mL/min；进样量为10 µL；洗脱程序：0～1 min，5%B；1～5 min，5%B～30%B；5～9 min，30%B～60%B；9～20 min，60%B～95%B；20～22 min，95%B；22～22.1 min，95%B～5%B；22.1～26 min，5%B；流动相A相为含0.2%甲酸的5 mmol/L乙酸铵水溶液，流动相B相为0.2%甲酸甲醇溶液。

#### 1.6.2 质谱条件

电喷雾离子源（ESI）；离子化模式：正离子；数据采集模式：多反应监测模式；气帘气压力（CUR）：0.21 MPa；喷雾电压（IS）：5 500.0 V；离子源温度（TEM）：650.0 ℃；雾化气压力（GS1）：0.41 MPa；辅助气压力（GS2）：0.38 MPa。54种目标化合物的质谱参数见表1。

**表 1:** 目标化合物的保留时间和质谱参数

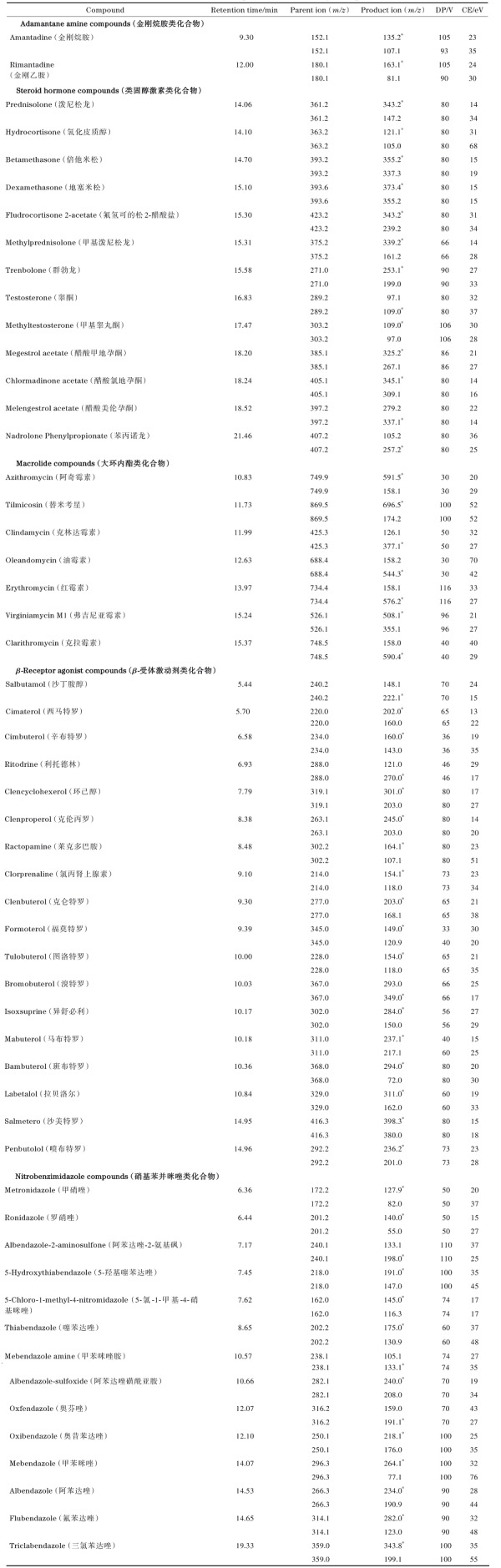

DP： declustering potential； CE： collision energy； * quantitative ion.

## 2 结果与讨论

### 2.1 磁性颗粒和ZIF-67的表征

按照1.3节和1.4节方法所制备的磁性四氧化三铁颗粒和ZIF-67的扫描电镜图如图1所示。由图1a可以看出，四氧化三铁颗粒的外观尺寸均匀，且结构与球形相似。同时，从图中可以观察到颗粒呈现出聚集分布的现象，这是因为四氧化三铁颗粒具有磁性的特性。由图1b可以看出，ZIF-67纳米材料呈十二面体结构，这是ZIF材料典型的晶体结构。由此可见，ZIF-67纳米材料已制备成功。

**图1:**
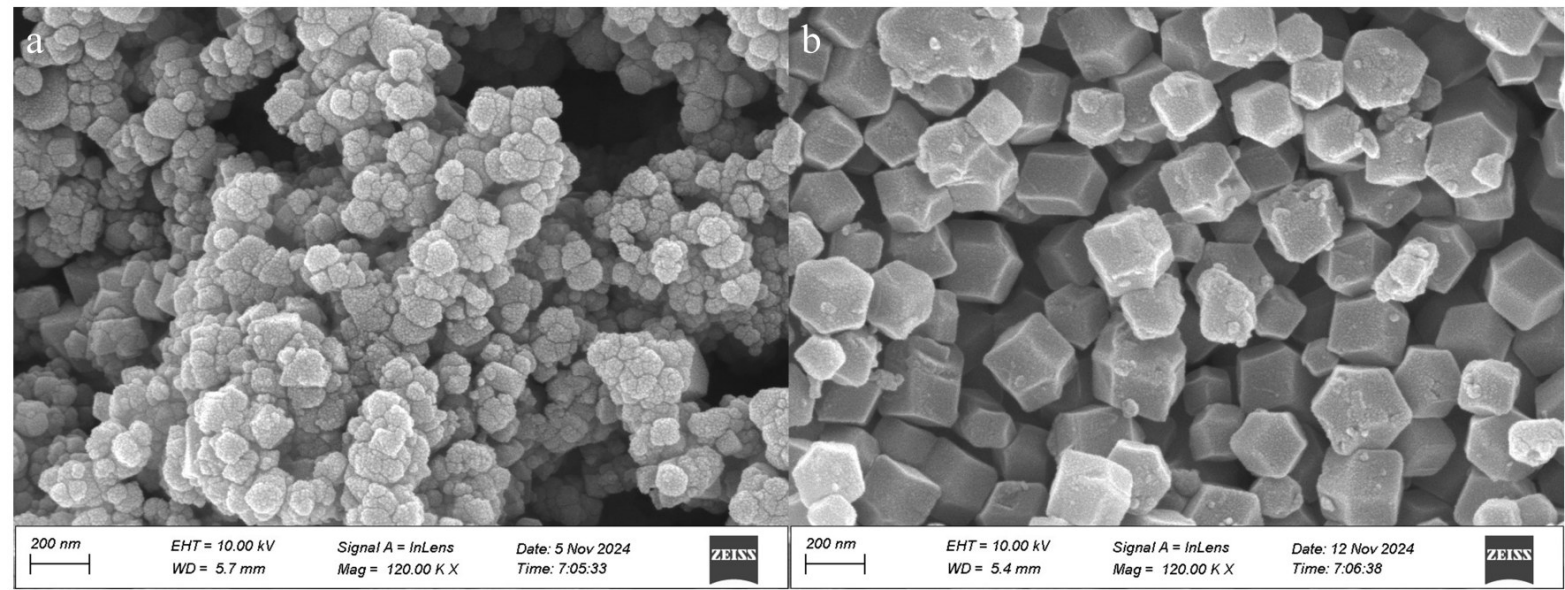
（a）磁性四氧化三铁颗粒和（b）ZIF-67的扫描电镜图

### 2.2 仪器分析条件的优化

在本研究中，我们利用HPLC-QTRAP-MS/MS对54种目标化合物进行了色谱分离和质谱定性定量分析。首先通过蠕动泵将目标化合物标准溶液注入ESI源，选用正/负离子切换全扫描模式对54种目标化合物进行全扫描，确定目标化合物母离子信息。实验结果表明，54种目标化合物均在正离子模式下的响应强度较高，更易形成准分子离子峰。再基于碎片离子扫描结果，选取响应强度相对较高的两个特征离子分别作为每一种目标物的定性和定量离子。54种目标化合物的质谱信息如表1所示。

采用C_18 _RRHD色谱柱（15 cm×4.6 mm，2.7 µm）对54种目标化合物进行色谱分离。实验结果表明，采用浓度为5 mmol/L的乙酸铵水溶液（含有0.2%甲酸）作为A相，0.2%甲酸甲醇作为B相，在梯度洗脱程序下，54种目标化合物的色谱峰具有较高的响应强度，其在MRM模式下的色谱图见图2。

**图2:**
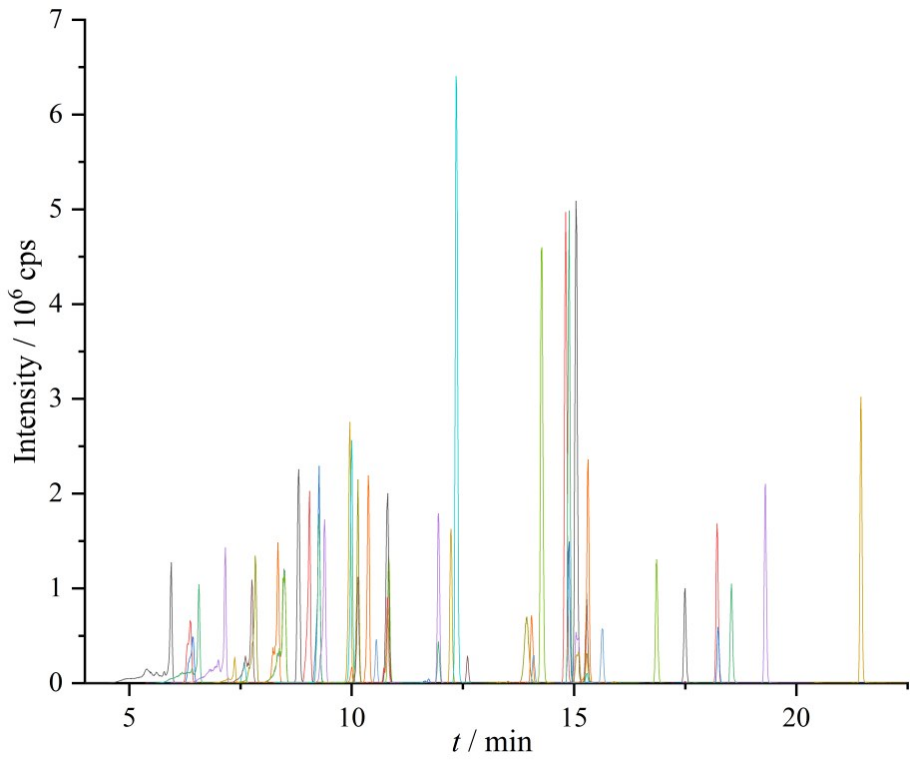
54种兽药混合标准溶液（10.0 μg/L）的MRM色谱图

### 2.3 样品前处理条件的优化

#### 2.3.1 净化材料质量的优化

为了降低干扰物质对检测结果的影响，本研究在上机前采用磁性一步式净化对样品提取液进行净化。ZIF-67表面含有氨基，可通过离子交换作用去除提取液中的脂肪酸和有机酸等干扰物质。本文比较了0、20、50、60、80和100 mg ZIF-67对54种兽药的净化效果，实验结果详见图3。结果表明，当使用60 mg ZIF-67作为净化材料时，样品提取液的净化效果最好，大部分目标物的回收率为80%~110%，因此最终选择60mg ZIF-67作为净化材料。

**图3:**
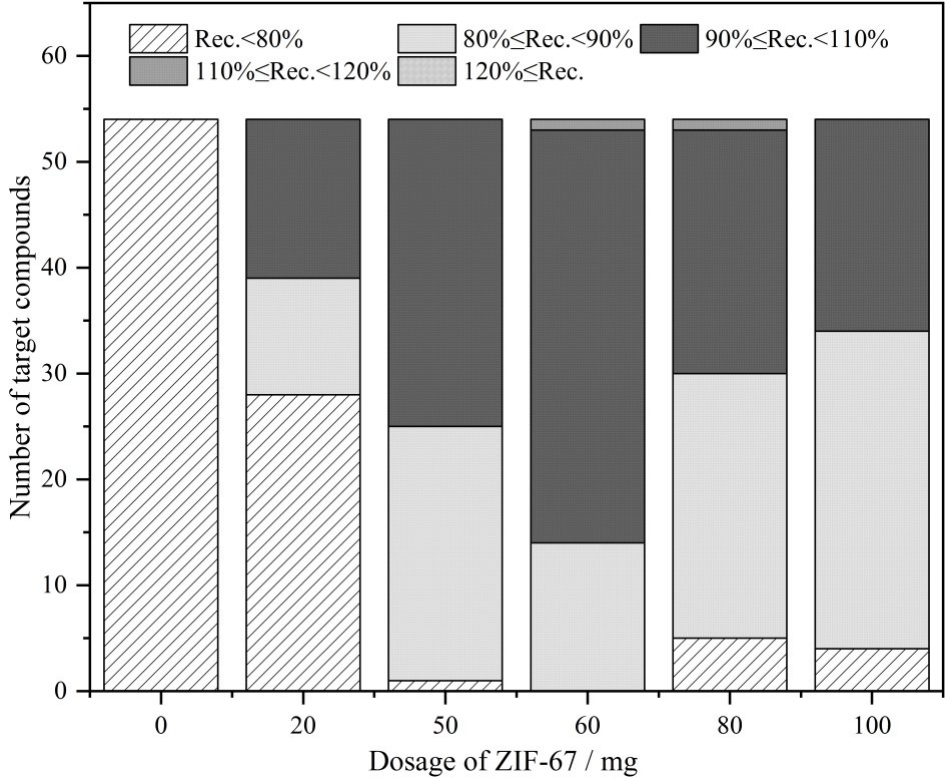
不同用量的ZIF-67对54种兽药净化效果的影响

#### 2.3.2 辅助净化材料质量的优化

C_18_是一种常用的除脂肪净化材料，其填料表面为疏水性的长链烷基基团，对提取液中的脂肪具有良好的吸附去除作用^［17］^。为了提高提取液中脂肪去除效率，本文在采用60 mg ZIF-67作为净化填料的基础上，进一步引入C_18_组成复合净化材料。本文详细比较了不同质量（0、20、50、80、100、120、150 mg）C_18_对54种兽药净化效果的影响，实验结果详见图4。实验结果表明，当使用60 mg ZIF-67和50 mg C_18_作为净化材料时，样品提取液的净化效果最好，大部分目标物的回收率为90%~110%，因此最终选择60 mg ZIF-67和50 mg C_18_作为净化材料组合使用。

**图4:**
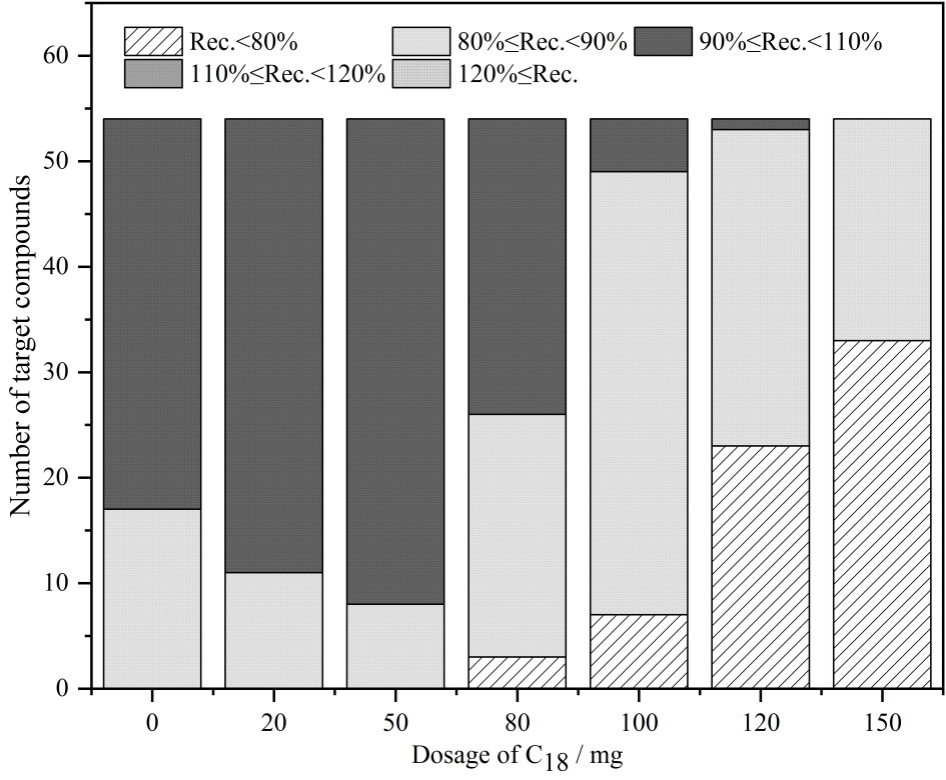
不同用量的C_18_对54种兽药净化效果的影响

### 2.4 基质效应

在使用质谱对水产品中的残留药物进行分析时，样品提取液中的干扰物质会影响目标化合物的质谱响应，进而产生基质效应（ME），并影响目标化合物定量结果的准确性^［18］^。在本研究中，为了评估基质效应对检测结果的影响，将ME定义为（*A*_m_/*A*_s_-1）×100%；其中，*A*_s_代表标准工作溶液的峰面积，*A*_m_代表基质匹配标准溶液的峰面积。实验结果表明，在54种化合物中，有37种化合物的ME小于0，表现出基质抑制效应；其余17种化合物的ME大于0，表现出基质增强效应。经过前处理净化操作之后，54种化合物的ME均在-20%~20%范围内，表现为弱基质效应，基质效应最为明显的化合物的基质效应值仅为18.11%。为了提高定量分析结果的准确性，本实验选择基质匹配校正法，可以在较大程度上降低基质效应的影响。

### 2.5 方法学验证

#### 2.5.1 线性方程、检出限和定量限

用空白样品基质提取液对54种兽药的混合标准工作溶液进行稀释，配制成系列质量浓度（0.5、1、2、5、10、20、50 µg/L）的基质匹配混合标准溶液，上机检测。以目标化合物的质量浓度为横坐标，峰面积为纵坐标，绘制基质匹配标准曲线。结果表明，54种兽药在0.5~50.0 µg/L范围内线性关系良好，相关系数（*r* ^2^）均大于0.99。以阴性鲤鱼样品为基质进行加标，分别以信噪比（*S/N*）等于3和10的样品含量作为LOD和LOQ，结果显示，54种兽药的LOD和LOQ分别在0.5~1.0 µg/kg和1.0~2.0 µg/kg范围内（见表2）。

**表 2:** 54种兽药的回归方程、相关系数、检出限和定量限

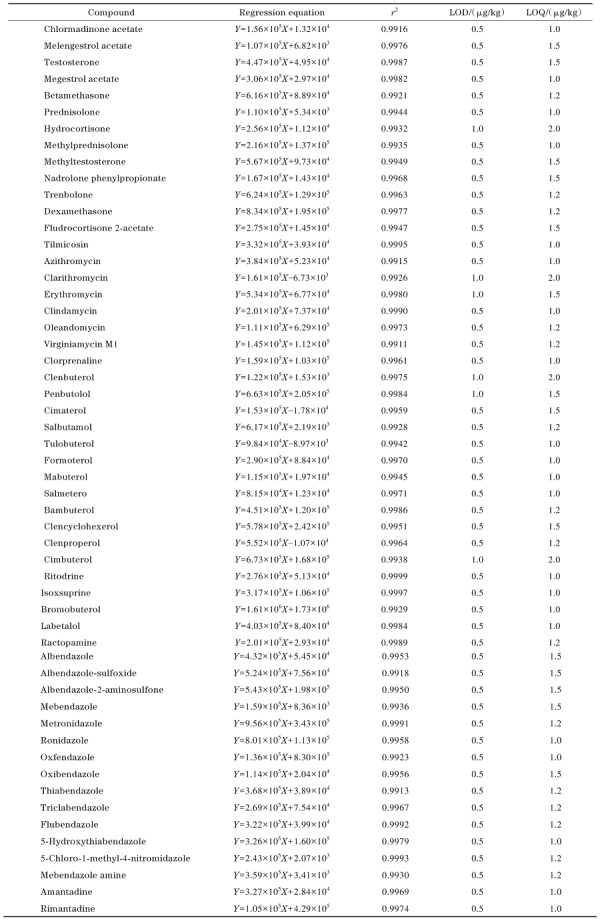

*Y*： peak area； *X*： mass concentration， µg/L.

#### 2.5.2 回收率和精密度

在空白鲤鱼基质中分别按照2.0、5.0、20.0 µg/kg 3个水平加入对应的混合标准溶液。每个添加水平设置6组平行进行测定，连续测定3天，计算加标回收率、日内相对标准偏差和日间相对标准偏差，具体结果详见表3。结果显示，在3个加标水平下，54种兽药的加标回收率为81.34%~109.85%，日内及日间相对标准偏差均小于10%，说明该方法准确度高，重复性好，能够满足水产品中兽药残留的检测需求。

**表3:** 54种兽药的加标回收率、日内和日间精密度

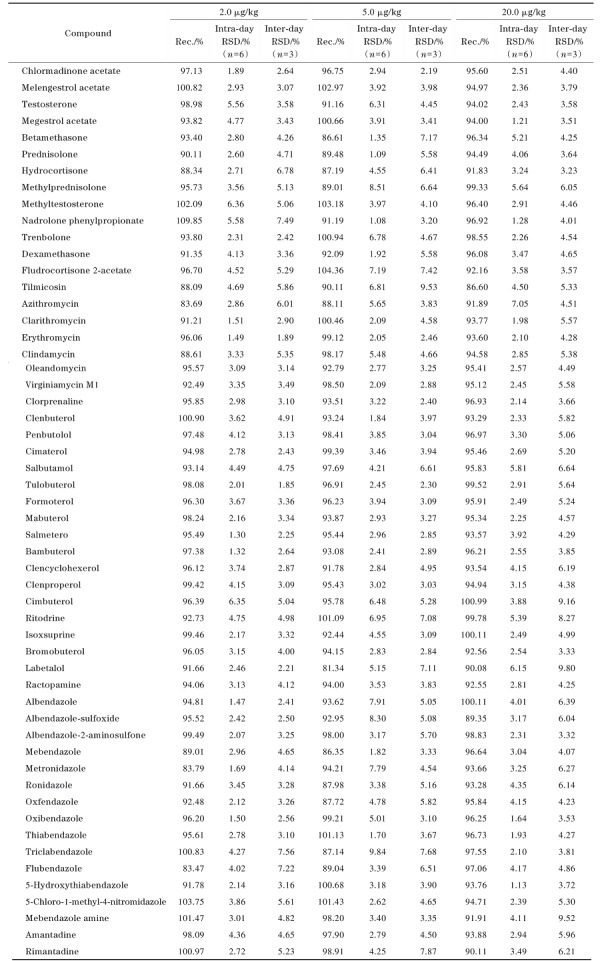

### 2.6 与其他方法的比较

为了评估该方法的快速性和准确性，本文将其与现有的兽药检测方法进行了系统比较，具体信息详见表4。在净化环节中，本研究通过引入磁铁实现了样品中杂质的快速高效分离。与传统方法相比，本方法有效解决了操作耗时较长的问题，通过省略离心等复杂步骤，将净化时间显著缩短至2 min以内。此外，磁性净化材料的应用不仅提升了分离效率，还简化了操作流程，减少了目标化合物在净化过程中的损失。因此，本方法在目标化合物回收率和灵敏度方面均表现出显著优势。同时，本研究成功克服了传统方法中吸附剂难以回收的问题，磁性颗粒的可重复利用特性提高了实验的环保性和经济性。综上所述，本研究为传统兽药检测技术面临的挑战提供了更加高效、经济且可靠的解决方案。

**表4:** 与其他方法的比较

Number of veterinary drugs	Samples	Sorbent	Purification method	Purification time/min	Instrumental method	LOQ/（μg/kg）	Rec./%	Ref.
26	fish and shrimp	EMR-Lipid	QuEChERS	13	HPLC-MS/MS	0.2-2.6	70.5-109.6	［^19^］
11	pork	C_18_	QuEChERS	15	HPLC-MS/MS	0.6-30.0	70.2-110.3	［^20^］
10	fish and shrimp	Al_2_O_3_	SPE	20	HPLC-MS/MS	0.5-1.0	61.5-119.0	［^21^］
16	pork	PSA+C_18_	QuEChERS	12	HPLC-MS/MS	0.03-0.82	70.4-118.1	［^22^］
54	fish	Fe_3_O_4_+ZIF-67	modified QuEChERS	2	HPLC-QTRAP-MS/MS	1.0-2.0	81.34-109.85	this work

### 2.7 实际样品的检测

为了进一步验证本研究所建立的检测方法的可行性，对来自北京市市场随机抽取的14份鲤鱼实际样品的肌肉部分进行检测。结果显示，所检测的实际样品中未发现有目标兽药的残留。

## 3 结论

本研究基于磁性一步式净化和HPLC-QTRAP-MS/MS技术，建立了鲤鱼中54种兽药残留物的快速分析方法。这种方法利用磁性复合材料的独特特性简化净化过程，从而显著提高了残留物检测的速度和准确性。同时，这些复合材料具备高吸附能力和选择性，表现出优异的净化性能，进一步提高了检测效率。该方法方便快捷，经济高效，灵敏度高，为鱼类产品中兽药残留监控、检测方法开发等方面提供了良好的理论依据和技术指导。
